# Parkin occurs in a stable, non-covalent, ∼110-kDa complex in brain

**DOI:** 10.1111/j.1460-9568.2007.06000.x

**Published:** 2008-01

**Authors:** Cindy Van Humbeeck, Etienne Waelkens, Olga Corti, Alexis Brice, Wim Vandenberghe

**Affiliations:** 1 Department of Neurosciences, Division of Experimental Neurology, University of Leuven, and the Department of Neurology, University Hospital Gasthuisberg Leuven, Belgium; 2 Department of Molecular Cell Biology, Biochemistry Section, University of Leuven Leuven, Belgium; 3 INSERM U679, Département de Génétique, Cytogénétique et Embryologie, Fédération de Neurologie and Faculté de Médecine Pierre et Marie Curie, Groupe hospitalier Pitié-Salpêtrière Paris, France

**Keywords:** binding partners, blue native gel electrophoresis, mouse, mutations, Parkinson's disease

## Abstract

Mutations in the gene for parkin, a 52-kDa E3 ubiquitin ligase, are a major cause of hereditary Parkinson's disease (PD). *In vitro* studies have identified a large number of parkin-interacting proteins. Whether parkin exists as a monomer or as part of a stable protein complex *in vivo* is uncertain. Here we demonstrate that endogenous parkin occurs in a stable, non-covalent, ∼110-kDa complex in native extracts from mouse brain, heart and skeletal muscle, while monomeric parkin is undetectable. Partial denaturation experiments indicate that this complex is at least a tetramer. Reported parkin-binding partners do not show detectable association with the parkin complex on native gels. Upon overexpression in COS1, SH-SY5Y or CHO cells, parkin accumulates predominantly as a monomer, suggesting that the interactors required for complex formation are available in limiting amounts in these cells. Importantly, PD-linked *parkin* mutations significantly impair parkin complex formation. These data demonstrate that parkin oligomerizes into a stable, non-covalent, heteromeric complex *in vivo*, and suggest that parkin may have as yet unidentified stable binding partners.

## Introduction

*Parkin* mutations are among the most common causes of familial Parkinson's disease (PD), accounting for approximately 50% of early onset autosomal recessive cases (Kitada *et al.*, 1998; Lücking *et al.*, 2000). Parkin is a cytosolic, 52-kDa E3 ubiquitin ligase responsible for targeting specific substrates for proteasomal degradation (Imai *et al.*, 2000; Shimura *et al.*, 2000; Zhang *et al.*, 2000). A variety of PD-linked *parkin* mutations have been shown to induce a loss of parkin function through direct catalytic impairment or by decreasing parkin solubility or stability (Henn *et al.*, 2005; Sriram *et al.*, 2005). Thus, pathogenic *parkin* mutations could cause neurodegeneration through a toxic accumulation of one or more parkin substrates. However, other PD-linked *parkin* mutations have no discernible impact on the intrinsic ubiquitin ligase activity, solubility or other biochemical properties of parkin (Hampe *et al.*, 2006), and the pathogenic mechanism of these mutations remains unknown.

A large number of putative parkin substrates have been reported, including cell division control-related protein 1 (CDCrel-1; Zhang *et al.*, 2000), synphilin-1 (Chung *et al.*, 2001), parkin-associated endothelin receptor-like receptor (Pael-R; Imai *et al.*, 2001), O-glycosylated α-synuclein (Shimura *et al.*, 2001), α/β-tubulin (Ren *et al.*, 2003), cyclin E (Staropoli *et al.*, 2003), the p38 subunit of the aminoacyl-tRNA synthetase complex (Corti *et al.*, 2003), the nuclear membrane-associated protein Ras-related nuclear small G-protein-binding protein 2 (RanBP2; Um *et al.*, 2006) and ataxin-2 (Huynh *et al.*, 2007). Importantly, parkin may also ubiquitinate substrates in proteasome-independent pathways (Lim *et al.*, 2005), as recently shown for epidermal-growth-factor receptor pathway substrate clone (Eps15; Fallon *et al.*, 2006), tumour necrosis factor receptor-associated factor 2 (TRAF2) and IκB kinase γ (IKKγ; Henn *et al.*, 2007).

In addition to binding transiently to substrates, parkin may also interact more stably with non-substrate-binding partners. For example, binding of parkin to C-terminus of heat shock protein 70 (Hsp70)-interacting protein (CHIP) has been reported to enhance the ubiquitin ligase activity of parkin (Imai *et al.*, 2002). Conversely, parkin activity is inhibited by interaction with bcl-2-associated athanogene 5 (BAG5; Kalia *et al.*, 2004) or with the chaperones Hsp70 (Imai *et al.*, 2002) or 14-3-3η (Sato *et al.*, 2006). According to a different study parkin functions in a multiprotein ubiquitin ligase complex including the F-box protein hSel-10 and cullin-1 (Staropoli *et al.*, 2003). In this complex hSel-10, rather than parkin itself, would be responsible for substrate recognition. Association with calcium/calmodulin-dependent serine protein kinase (CASK) may localize parkin to the synapse (Fallon *et al.*, 2002), while interaction with α/β tubulin may anchor parkin to microtubules (Yang *et al.*, 2005). Parkin may associate with the E2 enzyme ubiquitin-conjugating human enzyme 7 (UbcH7; Imai *et al.*, 2000; Shimura *et al.*, 2001), or may be attached to the proteasome through interactions with the proteasomal subunits regulatory particle non-ATPase 10 (Rpn10; Sakata *et al.*, 2003) or XAPC7 (Dachsel *et al.*, 2005). Finally, parkin has also been reported to interact with DJ-1 (Baulac *et al.*, 2004) and leucine-rich repeat kinase 2 (LRRK2; Smith *et al.*, 2005), two other proteins linked to familial PD.

Many of the reported parkin interactions were identified through *in vitro* assays, such as yeast-two-hybrid studies, and the *in vivo* significance of these interactions is uncertain. Whether parkin exists as a monomer or as part of a stable protein complex in the brain remains unknown. Here we address this question using velocity gradient centrifugation, blue native-polyacrylamide gel electrophoresis (BN-PAGE) and gel filtration chromatography.

## Materials and methods

### Antibodies

Polyclonal rabbit anti-parkin antibody (CS2132) was from Cell Signalling Technology; monoclonal parkin antibodies PRK8 and PRK109 (Pawlyk *et al.*, 2003) were a kind gift from Dr Virginia Lee (University of Pennsylvania); rabbit anti-CUL1 and rabbit anti-CASK were from Zymed; rabbit anti-hSel-10 (anti-hCdc4, 69-kDa isoform) was from Gentaur Molecular Products; mouse antibodies against CDCrel-1, UbcH7 and α-synuclein were from BD Transduction Laboratories; rabbit anti-Hsp70 was from Stressgen; polyclonal goat anti-DJ-1, rabbit anti-IKKγ (FL-419) and rabbit anti-TRAF2 (C-20) were from Santa Cruz Biotechnology; rabbit anti-BAG5 was from Exalpha Biologicals; mouse anti-α-tubulin was from Sigma; rabbit anti-α-tubulin and anti-β-tubulin and goat anti-synphilin-1 were from Abcam; rabbit anti-CHIP was from Calbiochem; rabbit anti-Rpn10 (anti-PSMD4) was from Abgent; rabbit anti-14-3-3η was from IBL; mouse anti-XAPC7 was from Biotrend; rabbit anti-p38 was a kind gift from Dr S. Kim (Seoul National University); and purified rabbit IgG was from Chemicon.

### Tissue extraction, glycerol gradient centrifugation and BN-PAGE

**Fig. 1:**
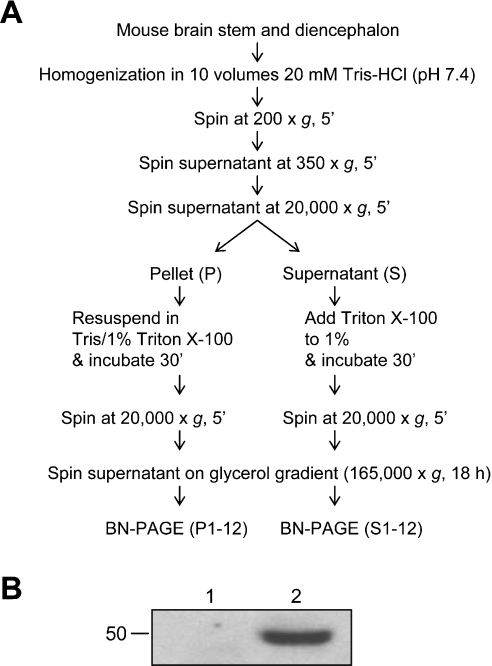
Tissue extraction, glycerol gradient centrifugation and blue-native-polyacrylamide gel electrophoresis (BN-PAGE) procedure. (A) Schematic overview of the procedure. (B) Mouse brain stem and diencephalon were homogenized as described in Materials and methods, and centrifuged at 350 ***g*** for 5 min. The pellet (Lane 1) and supernatant (Lane 2) obtained after the 350 ***g*** spin were compared for parkin content. SDS was added to the supernatant at a final concentration of 2%, and the pellet was resuspended in 2% SDS/20 mm Tris, pH 7.4. After boiling for 10 min and centrifugation at 14 000 ***g*** for 5 min, 60 µg of protein extract was loaded in each lane and examined by SDS–PAGE and Western blotting with the monoclonal anti-parkin antibody PRK109. The mobility of a 50-kDa molecular weight marker is shown on the left of the blot.

All the steps of tissue preparation, glycerol gradient centrifugation and BN-PAGE were performed at 4 °C. Brain stem and diencephalon, heart or skeletal muscle were dissected from 2- to 4-month-old wild-type (C57/Bl6J) or parkin-null (Itier *et al.*, 2003) mice after decapitation. Procedures followed were approved by the ethical committee of the University of Leuven. The tissue was homogenized with a glass pestle in 10 volumes of 20 mm Tris-HCl, pH 7.4, followed by trituration through a 200-µL pipette tip. The homogenization buffer did not contain salts, because salts may lead to precipitation of Coomassie dye and stained proteins on BN gels (Schägger *et al.*, 1994; Vandenberghe *et al.*, 2005a). Homogenates were centrifuged at 200 ***g*** for 5 min, followed by an additional 5 min spin at 350 ***g***(Fig. 1A). Control experiments indicated that the pellets obtained after the 200 ***g*** and 350 ***g*** centrifugation steps did not contain a significant amount of parkin (Fig. 1B). The supernatant obtained after the 350 ***g*** spin was centrifuged at 20 000 ***g*** for 2 min, leading to separation of pellet (P) and supernatant (S) fractions. P was resuspended in 10 volumes of 1% Triton X-100/20 mm Tris, pH 7.4. Triton X-100 was also added to S to a final concentration of 1%. After incubation for 30 min the samples were centrifuged at 20 000 ***g*** for 5 min. To test whether parkin was fully extracted in 1% Triton X-100, the pellets remaining after the final 20 000 ***g*** spin were solubilized in 1.5% sodium dodecyl sulphate (SDS) at 100 °C for 10 min. However, SDS–PAGE and Western blot with PRK8 did not detect any parkin in this Triton X-100-resistant, SDS-extractable fraction (data not shown). Protein concentrations of the Triton X-100 extracts were determined for P and S with the Bio-Rad Protein assay. Approximately 2 mg of protein extract was layered onto 10–50% glycerol gradients. Gradients were centrifuged in a SW-55 rotor (Beckman) at 4 °C for 18 h at 165 000 ***g***_av_ (Vandenberghe *et al.*, 2005a). Twelve fractions of 450 µL were collected manually from the top, and 90 µL of each fraction was loaded on BN gels. BN-PAGE and immunoblotting were performed as previously described in detail (Vandenberghe *et al.*, 2005a), except that 4–20% gradient gels were used in the present study. Immunoreaction was visualized with ECL or ECL Plus (Pierce), and exposure of the membrane to Kodak X-OMAT film. Antibody supershift experiments were performed as previously described (Yang *et al.*, 2002; Vandenberghe *et al.*, 2005a).

### Gel filtration chromatography

Approximately 1 mg of S fraction, prepared as described above except that Triton X-100 was omitted, was applied to a Superdex 200 HR 16/50 column (Pharmacia). The column was pre-equilibrated and eluted with 50 mm NaCl/25 mm Tris-HCl, pH 7.4. Elution was at a flow rate of 1 mL/min at 4 °C. Fractions of 0.5 mL were collected and analysed with dot blot for parkin. Calibration standards, including thyroglobulin (669 kDa), ferritin (440 kDa), lactate dehydrogenase (140 kDa), bovine serum albumin (67 kDa), vitamin D-binding protein (55 kDa) and protein phosphatase 2A phosphatase activator (36 kDa) were separated on the column under the same conditions and detected by UV light detection at 280 nm.

### Cell cultures and transfection

COS1 cells were cultured as previously described for COS7 cells (Vandenberghe *et al.*, 2005b). CHO and HEK-293 cells were cultured in a 1 : 1 mix of Dulbecco's modified Eagle medium (DMEM) and F-12 nutrient mixture (Invitrogen), supplemented with l-glutamine (2.5 mm), HEPES (15 mm), non-essential amino acids (1%), penicillin (100 U/mL), streptomycin (100 µg/mL) and 10% foetal calf serum. SH-SY5Y cells were cultured in a 1 : 1 mix of DMEM and F-12 nutrient mixture, supplemented with l-glutamax (4 mm), non-essential amino acids (1%), penicillin (100 U/mL), streptomycin (100 µg/mL) and 15% foetal calf serum. COS1, CHO and HEK-293 cells were transiently transfected using Lipofectamine Plus (Invitrogen), as previously described for COS7 cells (Vandenberghe *et al.*, 2005b). We transfected 80 ng of cDNA per cm^2^ of COS1, CHO or HEK-293 cell culture, unless otherwise indicated. SH-SY5Y cells were transiently transfected with the Nucleofector system (Amaxa) using the T16 program according to the instructions of the manufacturer. Human wild-type parkin cDNA was kindly provided by Dr K. Winklhofer (Max-Planck-Institute for Biochemistry, Martinsried, Germany; Winklhofer *et al.*, 2003). In experiments comparing wild-type and pathogenic parkin variants, COS1 cells were transfected with cDNAs encoding HA-tagged versions of wild-type, A82E, K161N, K211N, R256C and R275W parkin. The cDNAs for these HA-tagged parkin versions were constructed as previously described (Hampe *et al.*, 2006). Under the transfection conditions used in this study, phase-contrast microscopy immediately before cell harvest revealed no observable toxicity of COS1 cells expressing wild-type or PD-linked parkin versions (not shown). This was confirmed by SDS–PAGE and Western blot experiments showing similar β-tubulin levels in COS1 cells transfected with the different parkin variants (not shown), thus assuring similar amounts of cellular input material. Two days after COS1, CHO and HEK-293 cell transfection, and 24 h after SH-SY5Y cell transfection, the medium was aspirated and the cells were washed in ice-cold phosphate-buffered saline (PBS). The cells were removed from the plate with a cell scraper, resuspended in 1% Triton X-100/20 mm Tris, and solubilized for 30 min at 4 °C. Insoluble material was removed by centrifugation at 20 000 ***g*** for 5 min. Total protein concentrations of the Triton X-100 extracts were determined with the Bio-Rad Protein assay, and 500 µg of protein extract was layered onto 10–50% glycerol gradients. Glycerol gradient centrifugation and BN-PAGE was performed in the same way as for the brain extracts. In each of the glycerol gradient and BN-PAGE experiments comparing parkin complex formation between wild-type and pathogenic variants, we also compared the soluble levels of wild-type and mutant parkin versions by parallel SDS–PAGE of 15 µg of the Triton-soluble protein extracts. After Western blotting, the density of scanned signals was measured with UN-SCAN-IT gel 6.1 software (Silk Scientific). For quantitative experiments, film exposure times below the saturation range were used, as determined by multiple exposure times and as confirmed by the UN-SCAN-IT saturation check.

### Production and purification of recombinant parkin

GST-tagged wild-type parkin was produced in *Escherichia coli*, batch purified with glutathione-Sepharose, eluted from the beads and dialysed as previously described (Hampe *et al.*, 2006). GST-parkin was quantified by comparison with a bovine serum albumin standard after SDS–PAGE and staining of the gel with Coomassie Blue, aliquoted and stored at −80 °C. To remove the GST tag, 2.5 µg of GST-parkin was again bound to glutathione-Sepharose for 1 h at 4 °C in 1% Triton X-100/PBS (pH 7.4). The glutathione-Sepharose-bound GST-parkin was washed three times in 1% Triton X-100/PBS, and three times in cleavage buffer (in mm: Tris-HCl, 50, pH 7.0; NaCl, 150; EDTA, 1; dithiothreitol, 1). GST-parkin was then cleaved on the beads by incubation with 5 units PreScission Protease (Amersham) for 4 h at 5 °C. Effective removal of the GST moiety was confirmed by determination of the molecular mass of recombinant parkin using SDS–PAGE and Western blot (not shown).

### Statistics

Statistical significance of differences was analysed with one-way anova and Student–Newman–Keuls test (SigmaStat 3.5, Systat). Differences were considered significant at *P* < 0.05. Values represent mean ± SEM.

## Results

### *Parkin is part of a stable, non-covalent, ∼110-kDa complex* in vivo

**Fig. 2:**
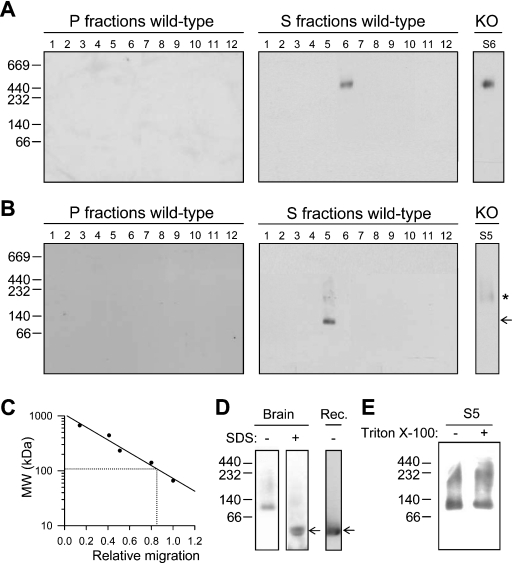
BN-PAGE reveals a stable, non-covalent, ∼110-kDa parkin complex in brain. (A and B) Extracts of brain stem and diencephalon from wild-type and parkin knockout (KO) mice were separated into pellet (P) and supernatant (S) fractions, as described in Materials and methods and Fig. 1A. P and S fractions were further fractionated by glycerol gradient centrifugation. Twelve P and 12 S fractions (numbered from the top of the glycerol gradient to the bottom) were analysed by BN-PAGE, followed by Western blotting with either the polyclonal anti-parkin antibody CS2132 (A) or the monoclonal anti-parkin antibody PRK109 (B) The CS2132 antibody detected a 450–550-kDa band in fraction S6, which was also found in parkin-null extract (A). By contrast, the PRK109 antibody (B) revealed a band in fraction S5 at a lower molecular weight (indicated by the arrow), which was absent in parkin knockout brain. The PRK109 antibody also showed some minor, non-specific immunoreactivity at higher molecular weights (indicated by the asterisk) in S5 from both wild-type and parkin knockout. (C) The graph shows how BN gels were calibrated based on the relative mobilities of native protein size markers to determine the molecular weight (MW) of the parkin complex. Markers, denoted by black circles, were thyroglobulin (669 kDa), ferritin (440 kDa), catalase (232 kDa), lactate dehydrogenase (140 kDa) and bovine serum albumin (67 kDa). The dotted line indicates the estimated MW of the parkin complex. (D) In the left and middle panels, heating brain fraction S5 at 100 °C in 1.5% sodium dodecyl sulphate (SDS) for 10 min immediately prior to BN-PAGE disrupted the ∼110-kDa parkin complex and led to the appearance of monomeric parkin (indicated by the arrow). In the rightmost panel, approximately 50 ng of purified recombinant parkin was analysed by BN-PAGE and Western blot with PRK109 without heat treatment or addition of SDS, revealing a band (indicated by the arrow) with apparent molecular weight (∼50 kDa) consistent with that of monomeric parkin. (E) Omission of Triton X-100 from the extraction protocol did not change the native molecular mass of the parkin complex from brain or the amount of parkin extracted. Numbers to the left of (A), (B), (D) and (E) indicate MWs of the native protein size markers.

In contrast to SDS–PAGE, BN-PAGE is performed in non-denaturing conditions and thus permits separation of intact non-covalent protein complexes (Schägger *et al.*, 1994; Vandenberghe *et al.*, 2005a). We used this technique to determine the native molecular mass of parkin extracted from the brain stem and diencephalon of 2- to 4-month-old mice. Brain homogenates were separated by centrifugation into pellet (P) and supernatant (S) fractions (Fig. 1A). Fraction P was enriched in membranes, as demonstrated by Western blotting for the α-amino-3-hydroxy-5-methyl-4-isoxazolepropionic acid (AMPA) receptor subunit glutamate receptor subunit 2 (GluR2), a neuronal membrane protein; fraction S was mostly cytosolic, as indicated by Western blotting for the cytoplasmic protein α-tubulin (data not shown). After incubation with 1% Triton X-100, solubilized proteins from fractions P and S were further fractionated by centrifugation on 10–50% glycerol gradients (Fig. 1A). The resulting fractions (P1–12 and S1–12) were subjected to BN-PAGE followed by immunoblotting with CS2132, a commonly used, commercially available polyclonal anti-parkin antibody. This antibody detected a band in fraction S6 with a very high molecular mass (450–550 kDa; Fig. 2A). To check the specificity of the CS2132 antibody, we performed parallel experiments on brain extracts from parkin-null mice. However, the 450–550-kDa complex was equally detected in parkin-null extracts, indicating that it was not an authentic parkin complex (Fig. 2A). The observed lack of specificity of the CS2132 antibody was in agreement with the findings of Pawlyk *et al.* (2003).

We therefore switched to using PRK8 and PRK109, two monoclonal antibodies that were previously demonstrated to be parkin specific (Pawlyk *et al.*, 2003). These antibodies detected a single band concentrated in fraction S5, which represented authentic parkin because it was absent in parkin-null extracts (Fig. 2B). This native parkin band had an estimated molecular mass of ∼110 kDa, based on its mobility relative to native markers of known molecular mass (Fig. 2C; Lee *et al.*, 2002). A small amount of parkin of similar molecular weight was detectable in fraction P5, but only after much more prolonged film exposure (data not shown). There was also some minor immunoreactivity at higher molecular weights (asterisk in Fig. 2B), but this was also found in the parkin knockout and thus probably resulted from non-specific binding. Remarkably, monomeric parkin was undetectable in native brain extracts (Fig. 2B). Importantly, purified recombinant parkin had an apparent molecular mass of ∼50 kDa on native gels (Fig. 2D), indicating that the ∼110-kDa parkin band represented a protein complex rather than an unusual tertiary conformation of the parkin monomer. Boiling brain extracts in 1.5% SDS for 10 min before BN-PAGE completely disassembled the ∼110-kDa parkin complex and led to the appearance of monomeric parkin (Fig. 2D), indicating that the parkin complex was held together by non-covalent interactions.

In the experiments shown in Fig. 2A–C brain extracts were solubilized in 1% Triton X-100. Although this is a mild detergent, we wondered whether any parkin interactions might still be disrupted. However, this was not the case, as shown by the fact that omission of Triton X-100 from the extraction protocol did not affect the apparent molecular mass of the parkin complex on native gels (Fig. 2E). Omission of Triton X-100 also did not diminish the amount of parkin extracted (Fig. 2E), consistent with the previous finding that mouse parkin is highly soluble and easily extracted from brain even in the absence of detergents (Pawlyk *et al.*, 2003).

**Fig. 3:**
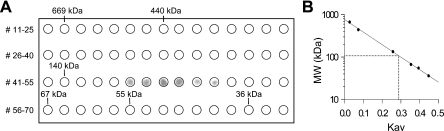
Demonstration of the parkin complex in brain by gel filtration chromatography. (A) Fraction S prepared in the absence of detergents was applied to a gel filtration column. Fractions of 0.5 mL were eluted from the column and fractions 11–70 were analysed with dot blot for parkin using the PRK109 antibody. Fraction numbers are indicated to the left of the blot. Molecular weights (MWs) indicate the elution patterns of the protein size markers thyroglobulin (669 kDa), ferritin (440 kDa), lactate dehydrogenase (140 kDa), bovine serum albumin (67 kDa), vitamin D-binding protein (55 kDa) and protein phosphatase 2A phosphatase activator (36 kDa). (B) Calibration graph of the gel filtration column. The black circles denote the protein size markers. *K*_av_ = (*V*_e_ − *V*_o_)/(*V*_t_ − *V*_o_), where *V*_e_ = elution volume, *V*_o_ = void volume and *V*_t_ = total column volume. The dotted line indicates the estimated MW of the parkin complex.

As an independent approach, we determined the molecular mass of native parkin by gel filtration chromatography of fraction S in the absence of detergents. Parkin eluted from the column as a single peak with an estimated molecular weight of ∼105 kDa (Fig. 3), consistent with the BN-PAGE results. Monomeric parkin was again undetectable.

**Fig. 4:**
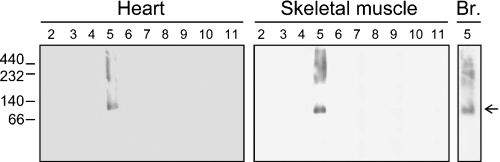
Formation of the ∼110-kDa parkin complex is conserved across tissues. S fractions were prepared from mouse heart, skeletal muscle and brain (Br.). The samples were analysed with glycerol gradient centrifugation, BN-PAGE and Western blotting with the monoclonal anti-parkin antibody PRK8. The arrow indicates the position of the ∼110-kDa parkin complex.

The *parkin* gene is not only expressed in brain but also in other tissues, especially heart and skeletal muscle (Kitada *et al.*, 1998). To find out whether the components of the ∼110-kDa parkin complex were also expressed outside the brain, we applied the protocol shown in Fig. 1A to mouse heart and skeletal muscle. The apparent molecular mass of parkin on native gels was similar in extracts from heart, skeletal muscle and brain (Fig. 4), suggesting that none of the components of the parkin complex was brain specific.

Next we performed immunoprecipitation experiments on brain extracts using the parkin-specific monoclonal antibodies and separated the immunoprecipitates by SDS–PAGE, in order to identify the components of the parkin complex by silver staining of the gels and mass spectrometry. However, comparison of silver-stained gels of immunoprecipitates from wild-type and parkin-null brains failed to reveal any specific bands (data not shown). This was possibly due to low abundance of the parkin complex in brain, low affinity of the monoclonal parkin antibodies, or masking of specific bands on the gel by the light or heavy chain bands of the immunoprecipitating antibody.

**Fig. 5:**
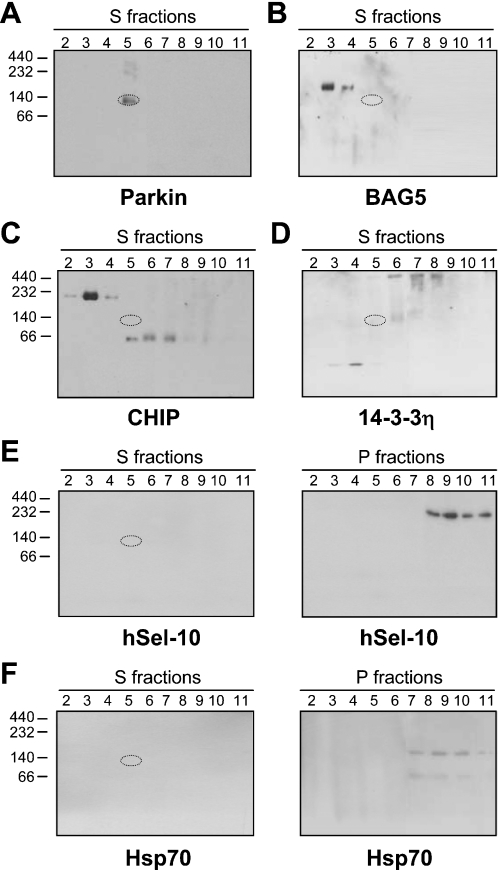
Known parkin-interacting proteins do not show detectable association with parkin on native gels. (A–F) S and P fractions from brain were separated by glycerol gradient centrifugation and BN-PAGE, followed by Western blotting for the protein indicated. The dotted ellipses indicate the position of the parkin band, as determined by reprobing each blot with anti-parkin.

**Fig. 6:**
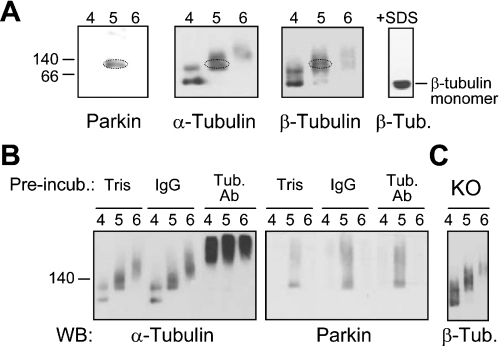
Lack of a detectable stable complex of parkin with tubulin. (A) Fraction S from brain was separated by glycerol gradient centrifugation, and fractions S4–S6 were analysed by BN-PAGE and Western blotting for parkin, α-tubulin or β-tubulin (β-Tub.). The dotted ellipses indicate the position of the parkin band, as determined by reprobing each blot with anti-parkin. In the rightmost panel, fraction S was boiled in 1.5% sodium dodecyl sulphate (SDS) for 10 min before BN-PAGE to identify the monomeric tubulin band. (B) Fractions S4–S6 were incubated for 90 min with 5 µg of control rabbit IgG, rabbit anti-α-tubulin (Tub. Ab), or buffer alone (Tris) before BN-PAGE. After Western blotting (WB) with mouse anti-α-tubulin (left panel), the blot was stripped and reprobed with mouse anti-parkin (right panel). Pre-incubation with polyclonal anti-α-tubulin fully shifted the α-tubulin bands to higher molecular weights due to antibody binding and cross-linking of tubulin complexes (left panel), but did not shift the parkin band (right panel). (C) The migration pattern of native β-tubulin from parkin knockout (KO) brain is similar to that in the wild-type extracts shown in (A).

As an alternative approach for identification of the components of the native parkin complex, we compared the migration of parkin in glycerol gradients and native gels with that of previously published parkin-binding partners. However, the reported parkin-interacting proteins BAG5 (Kalia *et al.*, 2004), CHIP (Imai *et al.*, 2002), 14-3-3η (Sato *et al.*, 2006), hSel-10 (Staropoli *et al.*, 2003) and Hsp70 (Imai *et al.*, 2002) migrated in characteristic, reproducible band patterns that clearly differed from that of parkin (Fig. 5). By contrast, immunoreactivity for α-tubulin and β-tubulin (Yang *et al.*, 2005), two proteins with monomeric molecular masses of ∼50 kDa, partially overlapped with the 110-kDa parkin band (Fig. 6A). One possible interpretation was that the parkin band represented a stable complex of parkin with monomeric tubulin. Alternatively, the overlap of immunoreactivity could simply be due to the close proximity of the molecular weight of the α/β-tubulin heterodimer to that of the parkin complex. To discriminate between these two possibilities, we performed antibody supershift experiments (Fig. 6B). If parkin and tubulin coexist in a stable complex, incubation of extracts with anti-tubulin antibody before BN-PAGE should change the migration of parkin due to antibody binding to parkin–tubulin complexes (Yang *et al.*, 2002; Vandenberghe *et al.*, 2005a). However, this was not the case, arguing against the existence of a stable parkin–tubulin complex in these extracts (Fig. 6B). This conclusion was further supported by the similar migration pattern of tubulin from wild-type and parkin-null brain extracts (Fig. 6C). Not unexpectedly, immunoreactivity for the 104-kDa protein CASK (Fallon *et al.*, 2002) also partially overlapped with the ∼110-kDa parkin band, but antibody supershift experiments and comparison with parkin-null extracts did not show evidence for the presence of a stable parkin–CASK complex (data not shown). Other published parkin-binding proteins, such as Rpn10 (Sakata *et al.*, 2003), XAPC7 (Dachsel *et al.*, 2005), UbcH7 (Imai *et al.*, 2000; Shimura *et al.*, 2001), cullin-1 (Staropoli *et al.*, 2003), DJ-1 (Baulac *et al.*, 2004), synphilin-1 (Chung *et al.*, 2001), CDCrel-1 (Zhang *et al.*, 2000), α-synuclein (Shimura *et al.*, 2001), p38 (Corti *et al.*, 2003), TRAF2 and IKKγ (Henn *et al.*, 2007), equally failed to show detectable association with the parkin complex on native gels (data not shown). We did not check the migration patterns of the recently reported parkin interactors LRRK2 (Smith *et al.*, 2005), RANBP2 (Um *et al.*, 2006), Eps15 (Fallon *et al.*, 2006) and ataxin-2 (Huynh *et al.*, 2007), because these proteins, with predicted molecular weights of ∼285 kDa, ∼385 kDa, ∼142 kDa and ∼136 kDa, respectively, were too large to be subunits of the ∼110-kDa parkin complex.

### The ∼110-kDa parkin complex is not a parkin homodimer

**Fig. 7:**
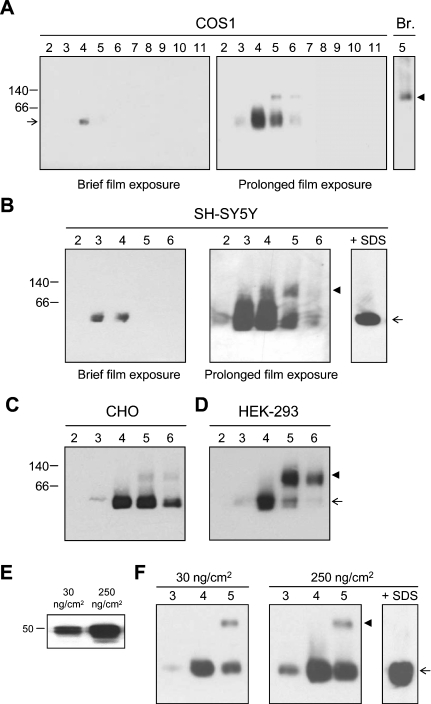
Overexpressed parkin accumulates predominantly as a monomer in several cell lines. (A) COS1 cells were transiently transfected with parkin and extracted in 1% Triton X-100, followed by glycerol gradient centrifugation of the extract. Glycerol gradient fractions 2–11 were analysed by BN-PAGE and immunoblotting for parkin. Upon overexpression parkin migrated as a monomer (indicated by the arrow on the left of the first panel). After more prolonged film exposure, a small amount of the ∼110-kDa complex was detected, as illustrated by comparison with the endogenous parkin band (indicated by the arrowhead) from brain (Br.). (B–D) SH-SY5Y (B), CHO (C) and HEK-293 (D) cells were transiently transfected with parkin and extracted in 1% Triton X-100. Glycerol gradient fractions 2–6 were analysed by BN-PAGE and immunoblotting for parkin. (B) The results of both brief and more prolonged film exposure are shown. In the rightmost panel of (B), total Triton-soluble extract from transfected SH-SY5Y cells was boiled in 1.5% SDS for 10 min immediately before BN-PAGE to identify the monomeric parkin band. The arrows and arrowheads indicate monomeric parkin and the ∼110-kDa parkin complex, respectively. (E) Either 30 or 250 ng of parkin cDNA was transfected per cm^2^ of COS1 cell culture. For each transfection condition, 20 µg of total protein extract was examined by sodium dodecyl sulphate (SDS)–PAGE and Western blotting, showing different total parkin expression levels. The mobility of a 50-kDa molecular weight marker is shown on the left of the blot. (F) Extracts of COS1 cells transfected with either 30 or 250 ng of parkin cDNA per cm^2^ were analysed by glycerol gradient centrifugation, and fractions 3–5 were subjected to BN-PAGE and parkin immunoblotting to visualize monomer (arrow) and ∼110-kDa complex (arrowhead). In the right panel, total Triton-soluble extract from COS1 cells transfected with 250 ng/cm^2^ of parkin cDNA was boiled in 1.5% SDS for 10 min immediately before BN-PAGE to identify the monomeric parkin band. When only 10 ng/cm^2^ of parkin cDNA was transfected, the protein was not detectable with the glycerol gradient centrifugation and BN-PAGE assay (not shown).

We considered the possibility that the ∼110-kDa parkin complex might be a parkin homodimer. However, this was highly unlikely for four reasons. First, the apparent molecular mass (∼50 kDa) of purified recombinant parkin on native gels (Fig. 2D) already suggested that parkin does not easily form homodimers. Second, parkin overexpression experiments in cell lines argued against a homodimeric composition of the parkin complex (Fig. 7). Endogenous parkin was not detectable with our BN-PAGE assay in untransfected COS1, SH-SY5Y, CHO or HEK-293 cells (data not shown). However, when we transfected parkin in COS1, SH-SY5Y or CHO cells, the protein accumulated predominantly as a monomer, with only minor formation of the ∼110-kDa complex (Fig. 7A–C). This strongly suggested that the ∼110-kDa complex was not a parkin dimer, but rather contained components that were present in limiting amounts in transfected COS1, SH-SY5Y and CHO cells. Interestingly, the abundance of the ∼110-kDa complex (relative to that of the parkin monomer) was dramatically higher in transfected HEK-293 cells (Fig. 7D) than in COS1, SH-SY5Y or CHO cells, suggesting that HEK-293 cells expressed higher endogenous levels of the binding partners required for complex formation.

Third, we also varied the transfection level of parkin in COS1 cells (Fig. 7E and F). With higher total parkin expression levels, there was relatively little change in the amount of complex, but there was a clear increase in the amount of monomer. Thus, formation of the ∼110-kDa complex again appeared to be limited by the endogenous levels of the required parkin-binding partners.

**Fig. 8:**
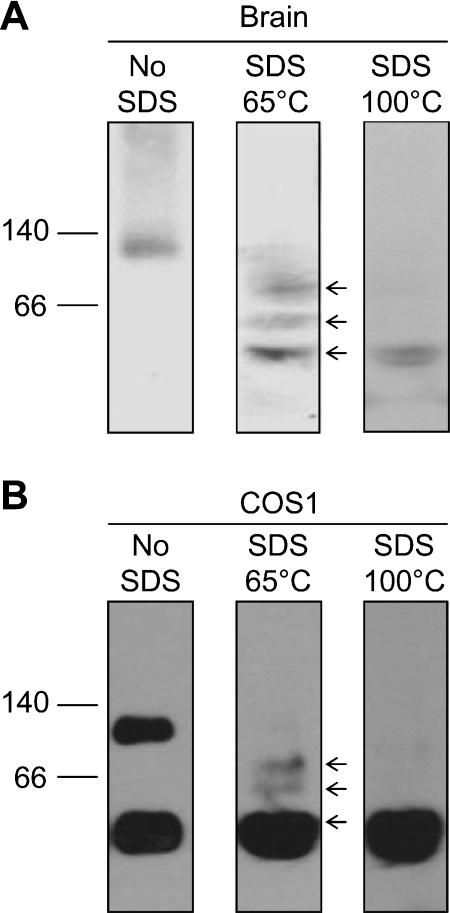
Partial dissociation of the parkin complex. (A) Heating fraction S from brain to 65 °C for 10 min in 1.5% sodium dodecyl sulphate (SDS) before BN-PAGE led to the appearance of three parkin bands (indicated by the arrows), suggesting that the ∼110-kDa complex was at least a tetramer. In the rightmost panel, fraction S was heated to 100 °C in 1.5% SDS for 10 min before BN-PAGE to dissociate the parkin complex completely and identify the position of the monomeric parkin band. (B) COS1 cells were transiently transfected with parkin and extracted in 1% Triton X-100. The cell extracts were treated in the same way as the brain extracts in (A).

Finally, partial denaturation experiments did not support a (homo)dimeric structure of the parkin complex. Heating extracts from brain (Fig. 8A) or transfected COS1 cells (Fig. 8B) to 65 °C (instead of 100 °C as in Fig. 2D) in 1.5% SDS led to partial dissociation of the parkin complex and the appearance of three parkin bands with estimated molecular masses of approximately 80, 60 and 50 kDa. The most parsimonious explanation for this observation would be that the ∼60-kDa band represented a complex of parkin with a protein other than parkin itself, the ∼80-kDa band reflected the association of a third component and the ∼110-kDa band arose from the addition of yet another subunit. According to this simple model, the ∼110-kDa complex would be a tetramer. Alternatively, it was also possible that, for example, the ∼60-kDa band represented a complex of parkin with more than one other protein, in which case the ∼110-kDa complex would contain more than four components. Hence, the findings suggested that the ∼110-kDa complex was at least a tetramer. In any case, the results shown in Fig. 8 were not consistent with the ∼110-kDa complex being a parkin homodimer.

### PD-linked parkin mutations compromise parkin complex formation

**Fig. 9:**
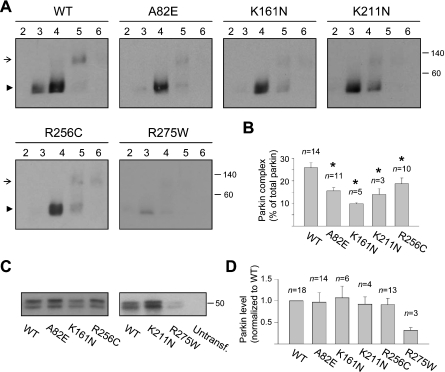
Comparison of ∼110-kDa complex formation between wild-type (WT) parkin and PD-linked parkin variants. COS1 cells were transiently transfected with 70 ng/cm^2^ of WT or R256C mutant parkin cDNA, 50 ng/cm^2^ of A82E or K161N parkin cDNA, 30 ng/cm^2^ of K211N cDNA and 120 ng/cm^2^ of R275W cDNA. (A) Transfected COS1 cells were extracted in 1% Triton X-100, followed by glycerol gradient centrifugation of the extracts. Fractions 2–6 of the gradient were analysed by BN-PAGE and parkin immunoblotting to visualize monomer (arrowhead) and ∼110-kDa complex (arrow). (B) In the experiments shown in (A), the amounts of parkin complex and parkin monomer were quantified. The graph represents the amount of parkin complex, expressed as a percentage of the sum of parkin complex and monomer. Asterisks denote significant difference (*P* < 0.05) from WT. (C and D) In parallel with each of the experiments shown in (A and B), 15 µg of Triton-soluble protein extract was analysed with SDS–PAGE and parkin immunoblotting to compare soluble parkin protein levels between WT and mutant variants. At the low signal intensity of the blots shown in (C), parkin appeared as a doublet due to the presence of an N-terminally truncated parkin species generated through an internal translation initiation site (Henn *et al.*, 2005). No endogenous parkin signal could be observed by SDS–PAGE in untransfected (Untransf.) COS1 cells (C), except after very prolonged film exposures (not shown). The graph in (D) shows SDS–PAGE and Western blot quantification of the Triton-soluble parkin levels. Within each experiment, the level of the parkin mutants was normalized to that of WT parkin. There were no significant differences in soluble parkin levels between WT, A82E, K161N, K211N or R256C (*P* = 0.96 by one-way anova).

Many pathogenic *parkin* mutations have been shown to cause a loss of parkin function by impairing its solubility, stability or catalytic activity (Henn *et al.*, 2005; Sriram *et al.*, 2005). However, a subset of PD-linked *parkin* mutations (including the A82E, K161N, K211N and R256C missense mutations) have little impact on parkin solubility, stability or interaction with known binding partners or on its intrinsic catalytic activity in a cell-free system (Hampe *et al.*, 2006). The pathogenic mechanism of these mutations thus remains elusive. We therefore explored whether the A82E, K161N, K211N and R256C mutations disrupted the ∼110-kDa complex. We transfected wild-type, A82E, K161N, K211N and R256C parkin cDNA into COS1 cells to determine the relative amounts of parkin complex and monomer. Importantly, Triton-soluble parkin levels were similar for the wild-type, A82E, K161N, K211N and R256C variants, as shown by parallel control experiments (Fig. 9C and D), thus precluding confounding effects of parkin expression levels on the observed complex/monomer ratios. Interestingly, each of the four mutations significantly impaired formation of the ∼110-kDa parkin complex (Fig. 9A and B). Quantitatively, the A82E, K161N, K211N and R256C mutations caused a ∼40%, ∼60%, ∼50% and ∼30% reduction, respectively, of the amount of parkin complex relative to total parkin (Fig. 9B). Given the similar soluble parkin expression levels (Fig. 9C and D), the differences in parkin complex/monomer ratio between wild-type and mutant proteins indicated a reduced intrinsic ability of the mutants to form the ∼110-kDa complex. We also examined the R275W parkin variant, which was previously shown to have reduced, but non-zero, solubility in Triton X-100 (Hampe *et al.*, 2006). The Triton-soluble level of R275W parkin was indeed lower than that of the other variants (Fig. 9C and D). A small amount of R275W parkin monomer was detected, but no complex was observed (Fig. 9A).

## Discussion

Mutations in the *parkin* gene are the most common known cause of autosomal recessive early onset PD (Lücking *et al.*, 2000). Seminal work has identified an E3 ubiquitin ligase function for parkin (Imai *et al.*, 2000; Shimura *et al.*, 2000; Zhang *et al.*, 2000). However, it is unclear whether parkin is a monomer or functions in a larger protein complex. To address this issue, we have investigated parkin under native conditions. Our study has led to two important conclusions. First, parkin exists in mouse brain and muscle as part of a stable, non-covalent, heteromeric complex with a molecular mass of ∼110 kDa. Surprisingly, monomeric parkin is not detectable *in vivo*. Second, PD-linked parkin mutations significantly compromise formation of this complex.

Recent work has shown that the solubility of parkin decreases in ageing human brain (Pawlyk *et al.*, 2003) and that oxidative stress can induce parkin aggregation (Winklhofer *et al.*, 2003; LaVoie *et al.*, 2005; Wang *et al.*, 2005). However, the ∼110-kDa parkin complex detected in this study is clearly not an aggregate, for three reasons. First, a parkin aggregate would be expected to be poorly soluble and to be extractable only in harsher detergents, whereas the ∼110-kDa parkin complex is highly soluble and easily extracted from mouse brain even in the absence of detergents. Second, aggregated parkin would be expected to appear on the gel as a ladder or smear of high-molecular-weight species rather than as a discrete band of ∼110 kDa. Third, conditions of transient overexpression generally increase the tendency of proteins to aggregate due to molecular crowding. However, upon overexpression in COS1, SH-SY5Y or CHO cells parkin accumulated in monomeric form with only limited formation of the ∼110-kDa parkin complex. This led to the conclusion that the ∼110-kDa complex was not an aggregate, but rather a complex of parkin with other proteins that were available in limiting amounts in the transfected cells.

In the present study previously reported parkin-interacting proteins did not show detectable association with the parkin complex after glycerol velocity gradient centrifugation and BN-PAGE. However, this assay is primarily designed to demonstrate interactions between stoichiometric components of stable protein complexes. The assay is less appropriate for detection of transient interactions (e.g. enzyme–substrate interactions) or of stable interactions that involve only a small percentage of the parkin molecules. Also, some parkin interactions may be weak and may therefore be disrupted during the prolonged centrifugation and electrophoresis procedures. Therefore, the lack of observable co-migration of the parkin complex and known parkin-binding proteins does not argue against the authenticity of the previously reported parkin interactions, and the present study by no means implies that parkin does not bind to its published partners. Nevertheless, our findings raise the tantalizing possibility that some of the most stable binding partners of parkin still remain to be identified.

It is tempting to speculate that the as yet unidentified constituents of the ∼110-kDa complex may be required for optimal parkin function. For example, the other subunits could control parkin stability, subcellular localization, substrate specificity or ubiquitin ligase activity (including the preference of parkin for mono- vs polyubiquitination). If so, genes encoding the other components of the complex would be attractive candidates for inherited forms of parkinsonism and other neurodegenerative syndromes. Intriguingly, we observed that the PD-linked parkin mutants A82E, K161N, K211N and R256C have a significantly reduced ability to form the ∼110-kDa complex. If complex formation is indeed necessary for proper functioning of parkin in cells, the observed reduced capacity for complex formation will explain the hitherto poorly understood pathogenic nature of these mutations (Hampe *et al.*, 2006). Also, this would resolve the paradox that the K161N and R256C mutants have less ubiquitination activity than wild-type when expressed in cells (Chung *et al.*, 2001; Sriram *et al.*, 2005), while possessing normal intrinsic ubiquitination activity in a cell-free system (Hampe *et al.*, 2006).

Interestingly, the absence of detectable monomeric parkin in brain and muscle suggests that endogenous expression levels of parkin are limiting for formation of this complex *in vivo*. This would make this complex exquisitely vulnerable to even slight reductions in the availability of soluble parkin that may result from parkin mutations (Winklhofer *et al.*, 2003; Henn *et al.*, 2005; Sriram *et al.*, 2005; Hampe *et al.*, 2006), as illustrated by our experiments with the R275W mutant. Cellular stress conditions (Winklhofer *et al.*, 2003; LaVoie *et al.*, 2005; Wang *et al.*, 2005) and normal ageing (Pawlyk *et al.*, 2003) are also known to impair parkin solubility and would therefore also be predicted to reduce the level of the parkin complex.

Further biochemical purification of the native parkin complex will be required to elucidate the identity of its components and to determine how they control parkin function. An analogy can be made with the identification of the subunits of the Alzheimer's disease-linked presenilin complex. One of the first indications that the presenilins functioned as part of a stable, oligomeric protein complex was that glycerol velocity gradient centrifugation suggested a much higher native molecular weight for the presenilins than expected based on their amino acid sequence (Capell *et al.*, 1998). It has taken several years and a variety of experimental approaches from multiple laboratories to identify nicastrin, anterior pharynx defective-1 (Aph-1) and presenilin enhancer-2 (Pen-2) as the additional constituents of the functional γ-secretase complex (De Strooper, 2003).

## Abbreviations

BAG5: bcl-2-associated athanogene 5
BN-PAGE: blue native-polyacrylamide gel electrophoresis
CASK: calcium/calmodulin-dependent serine protein kinase
CDCrel-1: cell division control-related protein 1
CHIP: C-terminus of Hsp70-interacting protein
DMEM: Dulbecco's modified Eagle medium
Eps15: epidermal-growth-factor receptor pathway substrate clone 15
Hsp70: heat shock protein 70
IKKγ: IκB kinase γ
LRRK2: leucine-rich repeat kinase 2
P: pellet
PBS: phosphate buffered saline
PD: Parkinson's disease
RanBP2: Ras-related nuclear small G-protein-binding protein 2
Rpn10: regulatory particle non-ATPase 10
S: supernatant
SDS–PAGE: sodium dodecyl sulphate–polyacrylamide gel electrophoresis
TRAF2: tumour necrosis factor receptor-associated factor 2
UbcH7: ubiquitin-conjugating human enzyme 7.
